# MGr1-Ag/37LRP induces cell adhesion-mediated drug resistance through FAK/PI3K and MAPK pathway in gastric cancer

**DOI:** 10.1111/cas.12414

**Published:** 2014-05-12

**Authors:** Li Sun, Lili Liu, Xiangqiang Liu, Yafang Wang, Mengbin Li, Liping Yao, Jianjun Yang, Genlin Ji, Changcun Guo, Yanglin Pan, Shuhui Liang, Biaoluo Wang, Jie Ding, Hongwei Zhang, Yongquan Shi

**Affiliations:** 1 State Key Laboratory of Cancer Biology, Xijing Hospital of Digestive Diseases, Fourth Military Medical UniversityXi'an, China; 2 Department of Oncology, Tangdu Hospital, Fourth Military Medical UniversityXi'an, China; 3 Department of Anesthesiology, Xijing Hospital, Fourth Military Medical UniversityXi'an, China

**Keywords:** CAM-DR, FAK/PI3K, gastric cancer, MAPK, MGr1-Ag/37LRP

## Abstract

It is well known that tumor microenvironment plays a vital role in drug resistance and cell adhesion-mediated drug resistance (CAM-DR), a form of *de novo* drug resistance. In our previous study, we reported that MGr1-Ag/37LRP ligation-induced adhesion participated in protecting gastric cancer cells from a number of apoptotic stimuli caused by chemotherapeutic drugs. Further study suggested that MGr1-Ag could prompt CAM-DR through interaction with laminin. However, the MGr1-Ag-initiated intracellular signal transduction pathway is still unknown. In this study, our experimental results showed that gastric cancer MDR cell lines mediated CAM-DR through upregulation of Bcl-2 by MGr1-Ag interaction with laminin. Further study found that, as a receptor of ECM components, MGr1-Ag/37LRP may activate the downstream signal pathway PI3K/AKT and MAPK/ERK through interaction with phosphorylated FAK. Moreover, the sensitivity to chemotherapeutic drugs could be significantly enhanced by inhibiting MGr1-Ag/37LRP expression through mAbs, siRNA, and antisense oligonucleotide. According to these results, we concluded that the FAK/PI3K and MAPK signal pathway plays an important role in MGr1-Ag-mediated CAM-DR in gastric cancer. MGr1-Ag/37LRP might be a potential effective reversal target to MDR in gastric cancer.

The resistance of gastric cancer (GC) cells to multiple chemotherapeutic agents remains a major obstacle in anticancer therapy. Although the problem of acquired drug resistance has classically been studied *in vitro*, drug resistance *in vivo* is still unsolved, mainly because these drug-resistant models lack consideration of the role of the tumor microenvironment. Increasing evidence suggests that the tumor microenvironment is the primary site leading to relapse after chemotherapy. Adhesion tumor cells to ECM components, such as fibronectin via β1-integrin, have been shown to confer resistance to a host of chemotherapeutic drugs.^1^,^2^ This anti-apoptotic phenomenon, called cell adhesion-mediated drug resistance (CAM-DR) is a form of de novo drug resistance.^3^–^5^ Therefore, identification of mediators of cell adhesion may elucidate novel targets for GC therapy and inhibition of these targets could potentially overcome CAM-DR.

Our laboratory previously reported MGr1-Ag as an upregulated protein in GC drug-resistant cell line SGC7901/VCR,^6^,^7^ and was identified as the 37-kDa laminin (LN) receptor precursor (37LRP).^8^ It has been shown to exhibit high laminin-binding activity, which is consistently observed in invasive and metastatic cancer cells and is associated with poor prognosis.^9^,^10^ We first reported that MGr1-Ag/37LRP may promote MDR of GC cells by decreasing intracellular drug accumulation and inhibiting drug-induced apoptosis.^11^ Further study suggested that MGr1-Ag could prompt CAM-DR through interaction with LN. However, the MGr1-Ag-initiated intracellular signal transduction pathway is still unknown.

Focal adhesion kinase (FAK) carries out protein–protein interaction adaptor functions at sites of cell attachment to the ECM, contributing to focal-adhesion “scaffolding”, and also transmits adhesion-dependent signals into the cell interior.^12^ Several studies have indicated that FAK has a direct role in tumor growth and survival by activating survival pathways of PI3K/AKT and MAPK/ERK.^13^,^14^

Here, we show that apoptosis induced by chemotherapeutic drugs may by partly inhibited by survival pathways of PI3-kinase/AKT and MAPK/ERK activated by the interaction of FAK and MGr1-Ag/37LRP after the adhesion of MGr1-Ag/37LRP to LN, which is MGr1-Ag/37LRP's ligand in the ECM of the gastric tumor microenvironment. We undertook these studies to characterize the role and the molecular mechanism of MGr1-Ag/37LRP in CAM-DR of GC cells to implicate a potential effective reversal target to MDR of GC.

## Materials and Methods

### Assessment of *in vivo* tumor growth

Approximately 1 × 10^6^ SGC7901/VCR cells were inoculated s.c. with 0.1 mL Matrigel (Sigma-Aldrich, St. Louis, MO, USA) in the flank region of 6–8-week-old male athymic nude mice (Experimental Animal Center, Fourth Military Medical University, Xi'an, China) using a 27-gauge needle under halothane anesthesia. When tumors reached 200 mm^3^, mice were randomly selected for treatment with vincristine alone (VCR), MGr1-Ag/37LRP antisense oligonucleotide (ASO) plus vincristine (VAS), scrambled ASO plus vincristine (VNS), MGr1-Ag/37LRP siRNA vector plus vincristine (VSM), scrambled RNA vector plus vincristine (VSP), mAb MGr1 plus vincristine (VAb), control antibody MGb2 plus vincristine (VIg), or bearing tumor without any treatment (TB). Each experimental group consisted of 10 mice. After randomization, 10 mg/kg MGr1-Ag/37LRP or scrambled ASO, 0.2 mg/kg MGr1-Ag/37LRP siRNA or scrambled RNA vector, and 100 mg/kg antibody MGr1 or MGb2 was injected i.p. once every 3 days for 36 days for treatment groups.^15^,^16^ A total of 0.6 mg/kg micellar vincristine was given i.v. three times per week from days 7 to 14 and from days 21 to 28.^17^ Tumor volume measurements were taken once every 4 days for 40 days and calculated by the formula length × width × depth × 0.5236 before being sampled.^18^ Data points were expressed as average tumor volume levels ±SE. All animal procedures were carried out according to the guidelines of the Chinese Council on Animal Care and with appropriate institutional certification. Half of the transplanted tumors in every group were dissected and fixed in formalin for immunohistochemical studies. Half of the tumors were also immediately harvested in cold isopentane, frozen in liquid nitrogen, and kept at −80°C for subsequent Western blot analysis.

### Statistical analysis

Each experiment was repeated at least three times. Numerical data are presented as the mean ± SEM. Analysis of variance was used to compare the differences between the experimental groups. The least significant difference *t*-test was used for multiple comparisons. All statistical analyses were done with the computer programs of spss 11.0 software (SPSS Inc., Chicago, IL, USA).

## Results

### Apoptosis induced by chemotherapeutic drugs was inhibited through Bcl-2 upregulated by MGr1-Ag interaction with LN

**Figure 1:**
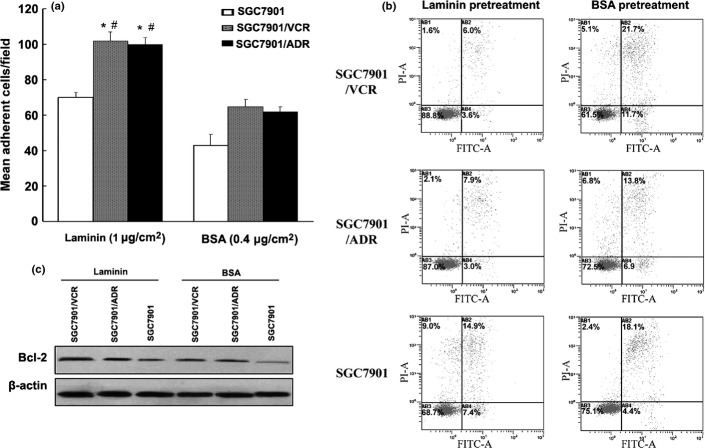
Role of Bcl-2 on the cell adhesion-mediated drug resistance phenotype in gastric cancer (GC) MDR variants cells. (a) Cell adhesion assay. After 2 h of adhesion, the cells attached to laminin (LN) and BSA were counted under a microscope. (b) Expression of Bcl-2 in SGC7901 cells in the condition of adhesion to LN (1 μg/cm^2^) and BSA (0.4 μg/cm^2^) was evaluated by Western blot. β-Actin was used as an internal control. (c) Apoptosis indexes of GC cells induced by vincristine (VCR) detected by flow cytometry. *P < 0.05 versus SGC7901 adhesion to LN; #*P *< 0.05 versus SGC7901/VCR and SGC7901/adriamycin (ADR) cell adhesion to BSA control. Representative experiments are shown from three for each cell type.

We previously reported that MGr1-Ag could prompt cell adhesion-mediated GC drug resistance through interaction with LN. It was reported that integrins mediated CAM-DR by upregulation of antiapoptotic Bcl-2.^19^ To gain insight into the mechanism of MGr1-Ag in CAM-DR, we first examined the adhesive potential of three GC cell lines, including two MDR GC cell lines SGC7901/VCR and SGC7901/adriamycin (ADR), as well as drug-sensitive cell line SGC7901, to LN. As shown in Figure1(a), SGC7901/VCR and SGC7901/ADR showed high adhesive potential to LN, whereas SGC7901 showed relatively low adhesive potential (Fig.1a). Next, we tested the Bcl-2 expression in GC cell lines adhesion to LN and control BSA. Western blot analysis revealed that the expression of Bcl-2 was increased in SGC7901/VCR and SGC7901/ADR cell lines' adhesion to LN than that of SGC7901 adhesion to LN and the two cell lines' adhesion to control BSA (Fig.1b). The annexin V–propidium iodide assay revealed that SGC7901/VCR and SGC7901/ADR cells, after adhesion to ECM components, showed significantly decreased apoptosis index values compared to that of control (Fig.1c). These results suggested that GC MDR cell lines mediated CAM-DR through upregulation of Bcl-2.

### Interaction of MGr1-Ag/37LRP and FAK in adhesion to ECM components

**Figure 2:**
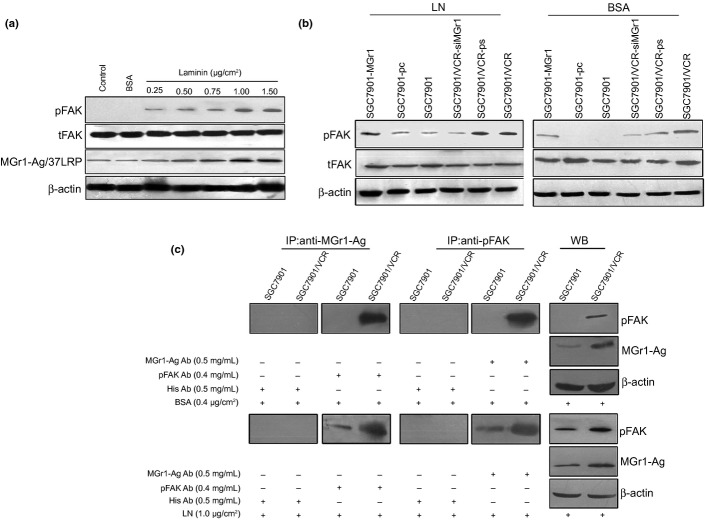
Characterization of the interaction between MGr1-Ag/37LRP and phosphorylated focal adhesion kinase (pFAK) in the condition of adhesion to laminin (LN). (a) Western blot analysis detected the expression of MGr1-Ag/37LRP, pFAK and total FAK (tFAK) in SGC7901 gastric cancer cells in the condition of adhesion to LN at the indicated concentration. (b) Western blot analysis detected pFAK and tFAK expression in transfected cells that expressed up- or downregulated MGr1-Ag/37LRP after adhesion to LN and BSA control. (c) Immunoprecipitation detected the interaction between MGr1-Ag/37LRP and pFAK in SGC7901/VCR and SGC7901 in adhesion to LN or control.

As FAK was a very important signal molecule associated with CAM-DR in many tumors, we first examined the phosphorylated FAK (pFAK) and total FAK (tFAK) expression in the SGC7901 cell adhesion to LN and BSA. Western blot analysis revealed expression of both MGr1-Ag/37LRP and pFAK at dose-dependent increases in SGC7901 induced by adhesion to LN at indicated concentration (Fig.2a). We then detected the pFAK and tFAK expression in the GC transfected cells that expressed up- or downregulated MGr1-Ag/37LRP after adhesion to ECM components and control. As shown in Figure2(b), the expression of pFAK in SGC7901-MGr1 after adhesion to LN significantly increased compared to that of SGC7901 and SGC7901-pc in the same conditions, and the expression of pFAK in SGC7901-MGr1 after adhesion to LN significantly increased compared to that of the same cells' adhesion to BSA. In addition, compared to the weak expression of pFAK in SGC7901-MGr1, the expression of pFAK could not be detected in SGC7901-pc or SGC7901 cells after adhesion to control. The expression of pFAK in SGC7901/VCR-siMGr1 after adhesion to ECM components and control significantly decreased compared to that of SGC7901/VCR and SGC7901/VCR-ps in the same conditions. As shown in Figure2(c), immunoprecipitation was recruited to evaluate the interaction between MGr1-Ag/37LRP and pFAK in the condition of adhesion to LN and control. Only in GC MDR variants SGC7901/VCR in adhesion to control, there was an interaction between MGr1-Ag/37LRP and pFAK. In adhesion to LN, there were different interactions between MGr1-Ag/37LRP and pFAK in both GC MDR variant SGC7901/VCR and drug-sensitive cells SGC7901. It was concluded that, as the receptor of ECM components, MGr1-Ag/37LRP may activate the downstream signal pathway through interaction with pFAK.

### Apoptosis induced by chemotherapeutic drugs was inhibited by Bcl-2 upregulated by the activated signal pathways PI3K/AKT and MEK/ERK1/2 induced by adhesion to LN

**Figure 3:**
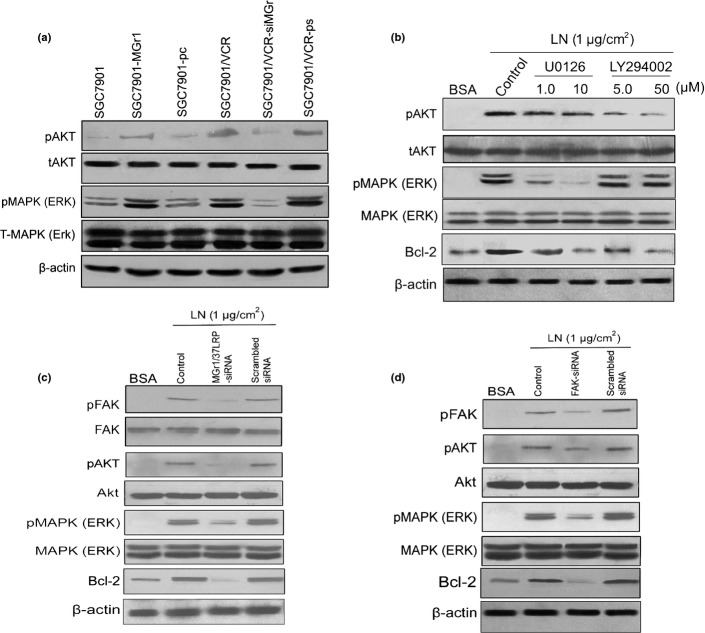
Effect of AKT and ERK1/2 activated by phosphorylated focal adhesion kinase (pFAK) on laminin (LN)- and exogenous MGr1-Ag/37LRP induced upregulation of Bcl-2. (a) Effect of exogenous MGr1-Ag/37LRP on AKT and ERK1/2 activation in gastric cancer (GC) cells that expressed up- or downregulated MGr1-Ag/37LRP. (b) Effect of adhesion to LN on AKT and ERK1/2 activation in GC cells. SGC7901 cells were cultured in 6-well plates coated by LN substrate (1 μg/cm^2^) or BSA as control for 4 h, followed by various doses of U0126 and LY294002 for 1 h. (c, d) SGC7901 cells were cultured in serum-free medium overnight, followed by transiently transfected MGr1-Ag or FAK siRNA expression plasmid (scramble sequence as control). The treated cells were then transferred into 6-well plates of LN substrate (1 μg/cm^2^) for 4 h. Cell lysates were subjected to Western blot analysis using antibodies against MGr1-Ag pFAK, total FAK, pAKT, total AKT (tAKT), pERK1/2, tERK1/2, Bcl-2, and β-actin. Representative experiments are shown from three for each cell type.

We found that LN- and exogenous MGr1-Ag/37LRP could induce phosphorylation of FAK in GC cells. As the survival pathways of PI3K/AKT and MEK/ERK1/2 could be activated by pFAK. We previously reported that MGr1-Ag overexpression could increase expression of Bcl-2 protein. So, we examined whether LN and exogenous MGr1-Ag/37LRP induced the upregulation of Bcl-2 through activation of the PI3K/AKT and MEK/ERK1/2 signaling pathways by Western blot analysis. As shown in Figure3, exogenous MGr1-Ag/37LRP and LN substrate could greatly increase AKT and ERK1/2 phosphorylation in SGC7901 cells. Transient transfection of cells with MGr1-Ag/37LRP or FAK siRNA partly blocked LN-induced AKT and ERK1/2 phosphorylation and inhibited LN-induced Bcl-2 expression. In addition, treatment with PI3K-specific inhibitor LY294002 and MEK1/2-specific inhibitor U0126 could block LN-induced AKT and ERK1/2 phosphorylation, respectively, and inhibited LN-induced Bcl-2 expression. It was concluded that LN- and exogenous MGr1-Ag/37LRP induced Bcl-2 expression may partly through signal pathway FAK-PI3K/Akt and MEK/ERK1/2.

### Sequence-specific and dose-dependent inhibition of MGr1-Ag/37LRP expression by mAb, ASO, and siRNA

**Figure 4:**
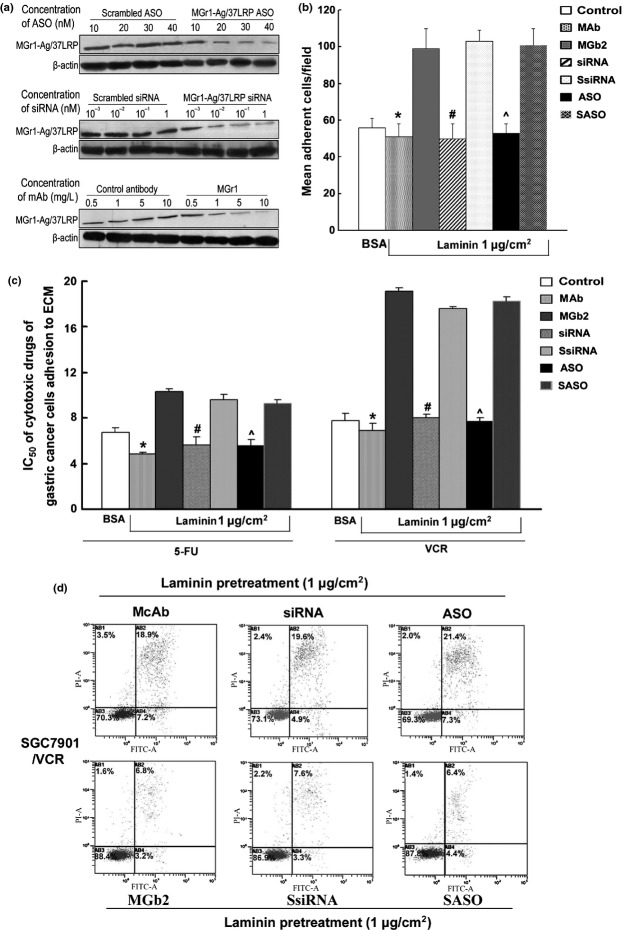
(On the previous page) Effect of MGr1-Ag/37LRP mAb, siRNA, and antisense oligonucleotide (ASO) treatment on gastric cancer (GC) drug-resistant cell line SGC7901/VCR tumor growth and chemosensitivity *in vitro*. (a) SGC7901/VCR cells were treated with indicated concentrations of MGr1-Ag/37LRP ASO or scrambled ASO (SASO) controls, MGr1-Ag/37LRP siRNA or scrambled siRNA (SsiRNA), and mAb MGr1 or control antibody for 48 h. Total protein was extracted from culture cells. (b) Cell adhesion assay. After 2 h of adhesion, the transfected cells and control cells attached to laminin or BSA was counted under a microscope. (c) Sensitivity of GC transfected cells to chemotherapeutic drugs was evaluated using the colony-forming assay. The concentration of each drug that caused a 50% reduction in the number of colonies (IC_50_) was calculated. (d) Apoptosis indexes of GC cells induced by vincristine (VCR) detected by flow cytometry. *P < 0.05 versus SGC7901/VCR cell incubated with control antibody; #*P *< 0.05 versus SGC7901/VCR scramble siRNA; ^*P* <  0.05 versus scramble ASO. Representative experiments are shown from three for each cell type.

To study the functional role of MGr1-Ag/37LRP in CAM-DR in GC, mAb-, ASO-, or siRNA-induced inhibition of MGr1-Ag/37LRP was determined by Western blot analysis. As shown in Figure4(a), treatment of SGC7901/VCR cells with ASO significantly reduced MGr1-Ag/37LRP protein levels by up to 75% in a dose-dependent manner, whereas MGr1-Ag/37LRP protein expression was not significantly suppressed by scrambled oligonucleotide. Similiar, MGr1-Ag expression was also inhibited at a dose-dependent manner using 10^−3^–1 nmol/L siRNA and 0.5–10 mg/L mAb. The cell adhesion assay, *in vitro* drug sensitivity assay, and annexin V/propidium iodide staining were used to exploit the MDR phenotype by blocking MGr1-Ag/37LRP with mAb, siRNA, ASO after adhesion to LN and control component (BSA) (Fig.4b–d). SGC7901/VCR cells transfected with siRNA (1 nmol/L), mAb (10 mg/L), and ASO (40 nM) showed significantly decreased mean adhesion cell number than that of control cells after adhesion to LN. Similarly, SGC7901/VCR cells transfected with siRNA (1 nmol/L), mAb (10 mg/L), and ASO (40 nM) showed significant increased IC_50_ values of VCR and 5-fluorouracil (5-FU) (Fig.4c), and decreased apoptotic index values in the same conditions (Fig.4d). These results suggested that inhibition of MGr1-Ag expression could partly reverse the CAM-DR phenotype *in vitro*.

### Monoclonal antibody, siRNA, and ASO for MGr1-Ag/37LRP can partly reverse MDR phenotype of SGC7901/VCR *in vitro* and *in vivo*

The colony-forming assay *in vitro* indicated that mAb (200 μg/mL), siRNA (0.4 μg/mL), and ASO (20 μg/mL) of MGr1-Ag/37LRP could decrease the IC_50_ values of VCR and 5-FU compared to that of the respective controls in SGC7901/VCR after adhesion to ECM components and BSA (Table1). The controls of mAb, siRNA, and ASO were MGb2 (200 μg/mL), scrambled siRNA (0.4 μg/mL), and scrambled ASO (20 μg/mL), respectively. Similar results were yielded by MTT assay (data not shown).

**Table 1:** Inhibitory concentration (IC_50_) values (mg/L, mean ± SEM) of gastric cancer MDR variants SGC7901/VCR in adhesion to ECM components and control for chemotherapeutic drugs 5-fluorouracil (5-FU) and vincristine (VCR) after treatment with mAb (200 μg/mL), antisense oligonucleotide (ASO; 20 μg/mL) and siRNA (0.4 μg/mL) for MGr1-Ag/37LRP

	5-FU			VCR		
	LN	COL IV	BSA	LN	COL IV	BSA
mAb	4.85 ± 0.36	5.01 ± 0.31	2.77 ± 0.63	6.91 ± 0.59	6.32 ± 0.28	5.17 ± 0.61
MGb2	10.30 ± 0.14*	11.14 ± 0.26*	6.21 ± 0.61*	19.12 ± 0.37*	17.73 ± 0.53*	7.59 ± 0.45*
siRNA	5.64 ± 0.27	4.57 ± 0.52	2.13 ± 0.34	8.02 ± 0.34	7.42 ± 0.66	5.49 ± 0.40
SsiRNA	9.62 ± 0.73**	10.25 ± 0.14**	5.33 ± 0.28**	17.65 ± 0.47**	18.34 ± 0.34**	7.98 ± 0.33**
ASO	5.56 ± 0.46	4.23 ± 0.31	2.85 ± 0.14	7.67 ± 0.22	6.67 ± 0.36	4.84 ± 0.51
SASO	9.25 ± 0.58***	11.30 ± 0.57***	6.11 ± 0.36***	18.28 ± 0.39***	17.24 ± 0.41***	8.55 ± 0.20***

The sensitivity of gastric cancer transfected cells to chemotherapeutic drugs was evaluated using a colony-forming assay. The concentration of each drug that caused a 50% reduction in the number of colonies (IC_50_) was calculated.

* *P *< 0.05 versus treatment with mAb.

** *P *< 0.05 versus treatment with siRNA.

*** *P *< 0.05 versus treatment with ASO. COL IV, collagen IV; LN, laminin; SASO, scrambled ASO; SsiRNA, scrambled siRNA.

**Figure 5:**
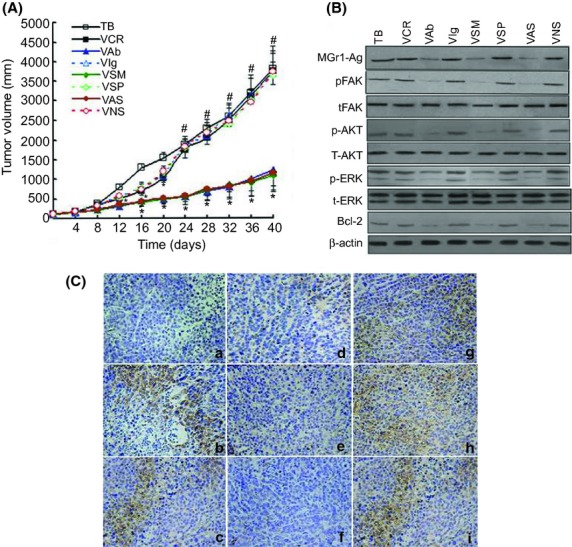
Effect of MGr1-Ag/37LRP mAb, siRNA, and antisense oligonucleotide (ASO) treatment on SGC7901/VCR tumor growth and chemosensitivity *in vivo*. (A) Mice bearing SGC7901/VCR tumors were randomly selected for treatment with vincristine (VCR) plus mAb (VAb), VCR plus siRNA (VSM), VCR plus ASO (VAS), VCR alone (VCR), VCR plus scrambled siRNA (VSP), VCR plus scrambled ASO (VNS), VCR plus control antibody (VIg), or the untreated group (TB). When SGC7901/VCR tumors reached 200 mm^3^, 0.2 mg/kg siRNA or scrambled siRNA, 100 mg/kg antibody MGr1 or control antibody, 10 mg/kg ASO or scrambled ASO were injected once every 3 days for 36 days. From days 7–14 and 21–28, 0.6 mg/kg VCR was given weekly by tail vein injection. Tumor volume was measured every 4 days and calculated by the formula: length × width × depth × 0.5236. Each point represents the mean tumor volume in each experimental group containing 10 mice. *P > 0.05 in each control group; #*P *< 0.05 versus respective controls, by least significant difference *t*-test. (B) Protein expression of MGr1-Ag/37LRP, phosphorylated focal adhesion kinase (pFAK), total FAK (tFAK), pAKT, tAKT, and Bcl-2 *in vivo*. Lysates were resolved by SDS-PAGE, and MGr1-Ag/37LRP, pFAK, tFAK, pAKT, tAKT, pERK, and tERK, Bcl-2 or β-actin (as indicated) was assessed by Western blot analysis. Representative experiments are shown from three for each cell type. (C) Immunohistochemical staining for MGr1-Ag/37LRP of paraffin-embedded sections from s.c. tumors: (a) negative control group; (b) TB group; (c) VCR group; (d) VAb group; (e) VSM group; (f) VAS group; (g) VIg group; (h) VSP group; (i) VNS group (original magnification, 200×). The results shown are representative of three independent experiments.

We then evaluated the effects of MGr1-Ag/37LRP mAb, siRNA, and ASO treatment on the chemotherapy of SGC7901/VCR tumors *in vivo*. Figure5(A) shows that therapy groups VCR plus mAb, VCR plus siRNA, and VCR plus ASO significantly reduced SGC7901/VCR tumor volume by 50% from days 16 to 40 compared to treatment with VCR alone, VCR plus control antibody, VCR plus scrambled siRNA, and VCR plus scrambled ASO, and the untreated group. In addition, after first treatment with VCR, from days 12 to 20, monotherapy of VCR significantly reduced SGC7901/VCR tumor volume compared to the untreated group. However, after first treatment with VCR, from days 24 to 40, there was no difference in SGC7901/VCR tumor volume between the VCR monotherapy group and the untreated group. Immunochemistry staining from transplant tumor tissue (Fig.5C) selected for therapy groups VCR plus mAb, VCR plus siRNA, and VCR plus ASO showed a remarkable decreased expression of MGr1-Ag/37LRP compared to the respective control groups. The expression of MGr1-Ag/37LRP and pFAK, p-AKT, p-ERK, and Bcl-2 *in vivo* were also detected in therapy groups of VCR plus mAb, VCR plus siRNA, and VCR plus ASO. In Figure5(B), Western blot analysis showed a remarkable decreased expression of MGr1-Ag/37LRP and inhibited the phosphorylation of FAK, AKT, and ERK, as well as subsequently downregulating expression of the Bcl-2 protein compared to the respective control groups. These data suggested that mAb, siRNA, and ASO, all of which target MGr1-Ag/37LRP, can enhance the sensitivity to chemotherapeutic drugs significantly in xenograft and inhibition of FAK, PI3K, and ERK might be another strategy to overcome CAM-DR.

## Discussion

The ECM, a complicated network composed of some multifunctional molecules, provides a sophisticated microenvironment for drug resistance.^20^,^21^ One of the main mechanisms underlying drug resistance has been ascribed to the adhesive property of GC cells to the ECM.^22^ It was reported that LN was a major component of basement membrane and it has been implicated in carcinogenesis and progression in GC cells.^23^ In our study, it was concluded that the adhesion ability of GC MDR variant cells was significantly increased compared to that of GC parental cells, which were more sensitive to chemotherapeutic drugs. After adhesion to the ECM component LN, the increased resistance to VCR and ADM suggested that the MDR phenotype of GC cells could be associated with the cell adhesion state.

Intercellular interactions may contribute to tumor cell survival during exposure to cytotoxic stresses such as chemotherapeutic drugs. It is also well documented that certain resistance mechanisms may only be functional *in viv*o, where tumor cells continue to interact with environmental factors such as the ECM and cellular counter-receptors. The signal transduction pathway causative for the CAM-DR phenotype has yet to be delineated. Activation of FAK occurs after external integrin-linked stimuli and starts autophosphorylation at tyrosine 397 either in an inter- or intramolecular manner, followed by recruitment of Scr-family kinase and binding and phosphorylates of MAPK.^24^ It was also reported that PI3K/AKT be activated by integrin^25^. Cell adhesion to fibronectin and phosphorylation of FAK, which is associated with α5β1 integrin and involved in cell survival signaling, were found to be increased in chemoresistant cells.^26^ Intracellular signal transduction cascades, PI3K/AKT and MEK/ERK, could also be initiated by FAK activated by formation of focal adhesion induced by integrin ligation to resist the toxicity of chemotherapeutic drugs in many tumors.^27^,^28^

It is well known that growth, differentiation, and progression of cancer cells are severely affected by the ECM. The role of LN on the progression of tumors has been intensively investigated. Gastric carcinomas have been reported to use α6β4 integrin and newly deposited laminins to adhere to surrounding tissues during invasion,^29^ and Koike *et al*.^30^ showed that invasive behavior of GC cells was inhibited by treatment with anti-α6 integrin antibody. Saito *et al*.^31^ reported that the production of MMP-9 by MKN1 human gastric carcinoma cells was potentiated by the α3β1 integrin–LN-5 interaction, which facilitated their invasion through degradation of the matrix. As 37LRP/67LR belongs to non-integrin adhesion molecular family, the alternation in the mechanisms of invasion or metastasis in GC need to be characterized. We previously reported that MGr1-Ag could induce CAM-DR through interaction with LN.

In this study, we found that MGr1-Ag interaction with LN could cause anti-apoptosis of acquired drug resistance in GC cells through upregulation of anti-apoptotic Bcl-2 expression. According to these results, the mechanisms of MGr1-Ag/37LRP ligation-initiated intracellular signal transduction pathways participated in protecting GC cells from a number of apoptotic stimuli caused by chemotherapeutic drugs. In our study, we also found that: (i) the expression of pFAK was upregulated in SGC7901-MGr1 cells pre-adhered to LN, compared with SGC7901-MGr1 pre-adhered to BSA; (ii) the expressions of pFAK and MGr1-Ag/37LRP were increased in a dose-dependent manner consistent with LN concentration; (iii) when cells were adhered to LN, pFAK interacted with MGr1-Ag/37LRP in SGC7901 and SGC7901/VCR cells, and this interaction was stronger in SGC7901/VCR; (iv) LY294002 could dose-dependently inhibit the phosphorylation of AKT in SGC7901 pre-adhered to LN, and U0126 could inhibit the phosphorylation of ERK1/2; and (v) Bcl-2 was decreased significantly with the downregulation of pAKT and pERK1/2. These results suggested that the ligation of MGr1-Ag/37LRP interaction with its ligand LN initiated FAK phosphorylation and thus activated PI3-K/AKT and MEK/ERK1/2 pathways in GC cells, and the downstream molecule Bcl-2 of both pathways was then upregulated and led to the resistance to apoptosis of adherent GC cells.

It was reported that the adhesion to its substrate could be blocked by β1 integrin antibody and subsequently could sensitize diverse tumor types, including GC to cytotoxic drugs.^3^,^32^,^33^ To further elucidate MGr1-Ag in CAM-DR, MDR reversal agents, ASO, mAb, and siRNA of MGr1-Ag/37LRP were used to address it. *In vitro,* MDR reversal agents, ASO, mAb, and siRNA of MGr1-Ag/37LRP could decrease the number of SGC7901/VCR cells adhering to LN and inhibition of MGr1-Ag could also cause apoptosis and reverse the CAM-DR phenotype. *In vivo*, MDR reversal agents, ASO, mAb, and siRNA of MGr1-Ag/37LRP could enhance the xenograft sensitivity to chemotherapeutic drug so significantly that the volume of transplanted tumor was decreased. The expression of MGr1-Ag/37LRP and phosphorylated FAK, AKT, ERK1/2, and Bcl-2 expression in xenograft tissues was decreased significantly after treatment with reversal agents. The same case for the expression of MGr1-Ag/37LRP in xenograft was revealed.

In conclusion, the present study disclosed that MGr1-Ag interaction with LN stimulates FAK, PI3K/AKT, and MEK/ERK signaling, thereby bringing cells into a primed state by which GC cells adhere to the ECM, resulting in the induction of CAM-DR. Hence, targeting the PI3K/AKT and MEK/ERK signal would be a promising strategy to overcome CAM-DR.

## References

[1] Hazlehurst LA, Damiano JS, Buyuksal I, Pledger WJ, Dalton WS (2000). Adhesion to fibronectin via beta1 integrins regulates p27kip1 levels and contributes to cell adhesion mediated drug resistance (CAM-DR). Oncogene.

[2] Matsunaga T, Fukai F, Miura S (2008). Combination therapy of an anticancer drug with the FNIII14 peptide of fibronectin effectively overcomes cell adhesion-mediated drug resistance of acute myelogenous leukemia. Leukemia.

[3] Noborio-Hatano K, Kikuchi J, Takatoku M (2009). Bortezomib overcomes cell-adhesion-mediated drug resistance through downregulation of VLA-4 expression in multiple myeloma. Oncogene.

[4] Damiano JS, Hazlehurst LA, Dalton WS (2001). Cell adhesion-mediated drug resistance (CAM-DR) protects the K562 chronic myelogenous leukemia cell line from apoptosis induced by BCR/ABL inhibition, cytotoxic drugs, and gamma-irradiation. Leukemia.

[5] Kobune M, Chiba H, Kato J (2007). Wnt3/RhoA/ROCK signaling pathway is involved in adhesion-mediated drug resistance of multiple myeloma in an autocrine mechanism. Mol Cancer Ther.

[6] Shi Y, Han Y, Wang X (2002). MGr1-Ag is associated with multidrug-resistant phenotype of gastric cancer cells. Gastric Cancer.

[7] Du J, Pan Y, Shi Y (2005). Overexpression and significance of prion protein in gastric cancer and multidrug-resistant gastric carcinoma cell line SGC7901/ADR. Int J Cancer.

[8] Shi Y, Zhai H, Wang X (2002). Multidrug-resistance-associated protein MGr1-Ag is identical to the human 37-kDa laminin receptor precursor. Cell Mol Life Sci.

[9] Annabi B, Currie JC, Bouzeghrane M (2006). Contribution of the 37-kDa laminin receptor precursor in the anti-metastatic PSP94-derived peptide PCK3145 cell surface binding. Biochem Biophys Res Commun.

[10] Jaseja M, Mergen L, Gillette K, Forbes K, Sehgal I, Copie V (2005). Structure-function studies of the functional and binding epitope of the human 37 kDa laminin receptor precursor protein. J Pept Res.

[11] Sun L, Shi Y, Guo C (2006). Regulation of multidrug resistance by MGr1-antigen in gastric cancer cells. Tumour Biol.

[12] McLean GW, Carragher NO, Avizienyte E, Evans J, Brunton VG, Frame MC (2005). The role of focal-adhesion kinase in cancer – a new therapeutic opportunity. Nat Rev Cancer.

[13] Diaz-Montero CM, Wygant JN, McIntyre BW (2006). PI3-K/Akt-mediated anoikis resistance of human osteosarcoma cells requires Src activation. Eur J Cancer.

[14] Kousidou O, Tzanakakis GN, Karamanos NK (2006). Effects of the natural isoflavonoid genistein on growth, signaling pathways and gene expression of matrix macromolecules by breast cancer cells. Mini Rev Med Chem.

[15] Burtrum D, Zhu Z, Lu D (2003). A fully human monoclonal antibody to the insulin-like growth factor I receptor blocks ligand-dependent signaling and inhibits human tumor growth in vivo. Cancer Res.

[16] Filleur S, Courtin A, Ait-Si-Ali S (2003). SiRNA-mediated inhibition of vascular endothelial growth factor severely limits tumor resistance to antiangiogenic thrombospondin-1 and slows tumor vascularization and growth. Cancer Res.

[17] Kuo CC, Hsieh HP, Pan WY (2004). BPR0L075, a novel synthetic indole compound with antimitotic activity in human cancer cells, exerts effective antitumoral activity in vivo. Cancer Res.

[18] Gleave M, Tolcher A, Miyake H (1999). Progression to androgen independence is delayed by adjuvant treatment with antisense Bcl-2 oligodeoxynucleotides after castration in the LNCaP prostate tumor model. Clin Cancer Res.

[19] de la Fuente MT, Casanova B, Garcia-Gila M, Silva A, Garcia-Pardo A (1999). Fibronectin interaction with alpha4beta1 integrin prevents apoptosis in B cell chronic lymphocytic leukemia: correlation with Bcl-2 and Bax. Leukemia.

[20] Timpl R (1996). Macromolecular organization of basement membranes. Curr Opin Cell Biol.

[21] Hohenester E, Engel J (2002). Domain structure and organisation in extracellular matrix proteins. Matrix Biol.

[22] Damiano JS, Cress AE, Hazlehurst LA, Shtil AA, Dalton WS (1999). Cell adhesion mediated drug resistance (CAM-DR): role of integrins and resistance to apoptosis in human myeloma cell lines. Blood.

[23] David L, Nesland JM, Holm R, Sobrinho-Simoes M (1994). Expression of laminin, collagen IV, fibronectin, and type IV collagenase in gastric carcinoma. An immunohistochemical study of 87 patients. Cancer.

[24] Hehlgans S, Haase M, Cordes N (2007). Signalling via integrins: implications for cell survival and anticancer strategies. Biochim Biophys Acta.

[25] Hazlehurst LA, Dalton WS (2001). Mechanisms associated with cell adhesion mediated drug resistance (CAM-DR) in hematopoietic malignancies. Cancer Metastasis Rev.

[26] Nakahara S, Miyoshi E, Noda K (2003). Involvement of oligosaccharide changes in alpha5beta1 integrin in a cisplatin-resistant human squamous cell carcinoma cell line. Mol Cancer Ther.

[27] Schwartz MA, Assoian RK (2001). Integrins and cell proliferation: regulation of cyclin-dependent kinases via cytoplasmic signaling pathways. J Cell Sci.

[28] Juliano RL (2002). Signal transduction by cell adhesion receptors and the cytoskeleton: functions of integrins, cadherins, selectins, and immunoglobulin-superfamily members. Annu Rev Pharmacol Toxicol.

[29] Tani T, Karttunen T, Kiviluoto T (1996). Alpha 6 beta 4 integrin and newly deposited laminin-1 and laminin-5 form the adhesion mechanism of gastric carcinoma. Continuous expression of laminins but not that of collagen VII is preserved in invasive parts of the carcinomas: implications for acquisition of the invading phenotype. Am J Pathol.

[30] Koike N, Todoroki T, Komano H (1997). Invasive potentials of gastric carcinoma cell lines: role of alpha 2 and alpha 6 integrins in invasion. J Cancer Res Clin Oncol.

[31] Saito Y, Sekine W, Sano R (2010). Potentiation of cell invasion and matrix metalloproteinase production by alpha3beta1 integrin-mediated adhesion of gastric carcinoma cells to laminin-5. Clin Exp Metastasis.

[32] Shain KH, Yarde DN, Meads MB (2009). Beta1 integrin adhesion enhances IL-6-mediated STAT3 signaling in myeloma cells: implications for microenvironment influence on tumor survival and proliferation. Cancer Res.

[33] Lin MT, Chang CC, Lin BR (2007). Elevated expression of Cyr61 enhances peritoneal dissemination of gastric cancer cells through integrin alpha2beta1. J Biol Chem.

